# Aryl Hydrocarbon Receptor Activates *NDRG1* Transcription under Hypoxia in Breast Cancer Cells

**DOI:** 10.1038/srep20808

**Published:** 2016-02-08

**Authors:** En-Yu Li, Wei-Yung Huang, Ya-Chu Chang, Mong-Hsun Tsai, Eric Y. Chuang, Qian-Yu Kuok, Shih-Ting Bai, Lo-Yun Chao, Yuh-Pyng Sher, Liang-Chuan Lai

**Affiliations:** 1 Graduate Institute of Physiology, National Taiwan University, Taipei, Taiwan; 2 Institute of Biotechnology, National Taiwan University, Taipei, Taiwan; 3 Bioinformatics and Biostatistics Core, Center of Genomic Medicine, National Taiwan University, Taipei, Taiwan; 4 Graduate Institute of Biomedical Electronics and Bioinformatics, National Taiwan University, Taipei, Taiwan; 5 Graduate Institute of Clinical Medical Science, China Medical University, Taichung, Taiwan; 6 Center for Molecular Medicine, China Medical University Hospital, Taichung, Taiwan

## Abstract

Hypoxia has been intensively investigated over the past several decades based on the observations that hypoxic tumors are more resistant to therapy and have a worse prognosis. Previously, we reported that N-myc downstream-regulated gene 1 (*NDRG1*) is strongly up-regulated under hypoxia and may play an important role in tumor adaptation to fluctuating oxygen concentrations. However, the regulatory mechanism of *NDRG1* under hypoxia remains elusive. Therefore, the purpose of this study was to identify the transcription factors that regulate *NDRG1* and to investigate the functional roles of *NDRG1* in hypoxia. We showed that binding sites of aryl hydrocarbon receptor (AHR) were predicted in the *NDRG1* promoter. Nuclear AHR was up-regulated in the presence of cobalt and hypoxia. AHR translocated to nuclei and bound between base pairs −412 and −388 of the *NDRG1* promoter in hypoxia. Moreover, hypoxia-mimetic induction of *NDRG1* was attenuated by knockdown of AHR expression. Also, overexpression of AHR facilitated cell proliferation and migration via up-regulation of *NDRG1*. These results showed for the first time that AHR positively regulates *NDRG1* transcription through an AHR binding site by way of hypoxia-mimetic signaling, which may lead to development of a specific therapeutic regimen to prevent tumor malignancy under hypoxia.

Breast cancer is one of the most common cancers, with more than 1.3 million cases each year worldwide^1^. This heterogeneous disease has distinct etiology, relapse risk, and therapy^2^. Moreover, microenvironmental hypoxia, a hallmark of solid tumors, is observed in breast cancer cells^3^. Since tumors grow rapidly and are provided with poor blood perfusion via inadequate vasculatures, hypoxia is widespread in both primary tumors and their metastases^4^,^5^. Although hypoxic conditions are harmful to cancer cells, hypoxia applies selective pressure for the tumor to adapt, thereby promoting malignant progression^6^,^7^.

Hypoxia affects DNA structure, gene expression, and signaling pathways and results in the induction of apoptosis, angiogenesis, glycolysis cascades, and cell cycle control^8^,^9^,^10^. These changes induce malignant cell survival and lead to undesirable outcomes. Furthermore, clinical studies also reveal that hypoxic tumors may become less sensitive to radio- and chemotherapy^11^. However, the exact relationship between hypoxia and tumor development remains unclear.

The transition metals, such as nickel and cobalt, have been widely used as hypoxia mimics in cell culture and as a tool to study the signaling cascades in the regulation of hypoxia-inducible gene expression^12^. Conveniently, nickel and cobalt can activate hypoxia-responsive signaling pathways under normoxia. These two transition metals are known to activate hypoxic signaling by stabilizing cytosolic hypoxia-inducible factor-1α (HIF-1α) via inhibiting the prolyl hydroxylase domain (PHD)-containing enzymes, a family of enzymes that play a key role in oxygen-dependent degradation of HIF-1α^13^.

N-myc downstream-regulated gene 1 (NDRG1) is a stress-responsive protein. The expression of *NDRG1* is triggered by multiple stimuli, including heavy metals (e.g., nickel, cobalt, and iron), hypoxia, oxidative stresses, DNA damage, and histone deacetylase inhibitors^14^,^15^. In response to these stimuli, NDRG1 plays roles in regulating cellular differentiation, proliferation, growth arrest, apoptosis, angiogenesis, tumor progression and metastasis, and heavy metal or hypoxia sensing^15^,^16^,^17^. In addition, *NDRG1* is up-regulated by a variety of hypoxia-related transcription factors, including HIF-1^18^, activating protein 1 (AP-1)^19^, early growth response 1 (EGR-1)^20^, and specificity protein 1 (SP1)^20^. Although hypoxia is a common feature of solid tumors that has been associated with tumor development and malignancy^6^,^7^, the role of *NDRG1* in these processes and the regulatory networks remains elusive.

Aryl hydrocarbon receptor (AHR), a ligand-activated transcription factor, is one member of the basic helix-loop-helix/Per-Arnt-Sim (bHLH/PAS) family, which mediates the biological effects of environmental pollutants, such as polycyclic aromatic hydrocarbons, planar polychlorinated biphenyls, and dioxins^21^,^22^. AHR is located in the cytosol as a protein complex with other proteins, such as the chaperone heat shock protein 90 (hsp90)^23^, and co-chaperone protein p23^24^. After ligand binding, AHR translocates to the nucleus and dimerizes with the aryl hydrocarbon nuclear translocator (ARNT)^25^. The AHR/ARNT heterodimers bind to the promoters of genes with a xenobiotic response element (XRE), 5′-GCGTG-3′, and subsequently induce transcription of downstream genes in the AHR pathway^26^. These genes encode xenobiotic-metabolizing enzymes, which are involved in biotransformation and detoxification of harmful xenobiotics^27^,^28^.

Similar to AHR, HIF-1α, a molecular sensor of oxygen tension, is also able to dimerize with ARNT. Under low oxygen conditions, HIF-1α translocates to the nucleus and forms a heterodimer with ARNT^29^. The HIF-1α/ARNT complex binds to the hypoxia response element (HRE), 5′-RCGTG-3′, and activates the transcription of genes involved in adaptation to hypoxia^30^. Since ARNT is important for both AHR and HIF-1α pathways, it seems possible that cross-talk exists between the two pathways. Some reports suggested that activation of one pathway leads to inhibition of the other due to the decreased availability of ARNT^31^,^32^,^33^; however, other studies revealed the existence of a compensation mechanism between the AHR and HIF-1 pathways^31^. Furthermore, recent studies suggested that AHR can act as a potential therapeutic target in multiple tumors^34^,^35^,^36^. However, the exact regulatory interactions between AHR and hypoxic tumors remain unclear.

Previously, we showed that *NDRG1* plays a role in tumor adaptation to the fluctuation of oxygen concentrations in MCF-7 breast cancer cells. This follow-up study is to investigate the *NDGR1* regulatory mechanism induced by hypoxia and its physiological functions. We found that *NDRG1* was up-regulated by AHR binding to its promoter, and that overexpression of AHR enhanced cell proliferation and migration under hypoxia through up-regulation of *NDRG1*.

## Results

**Figure 1:**
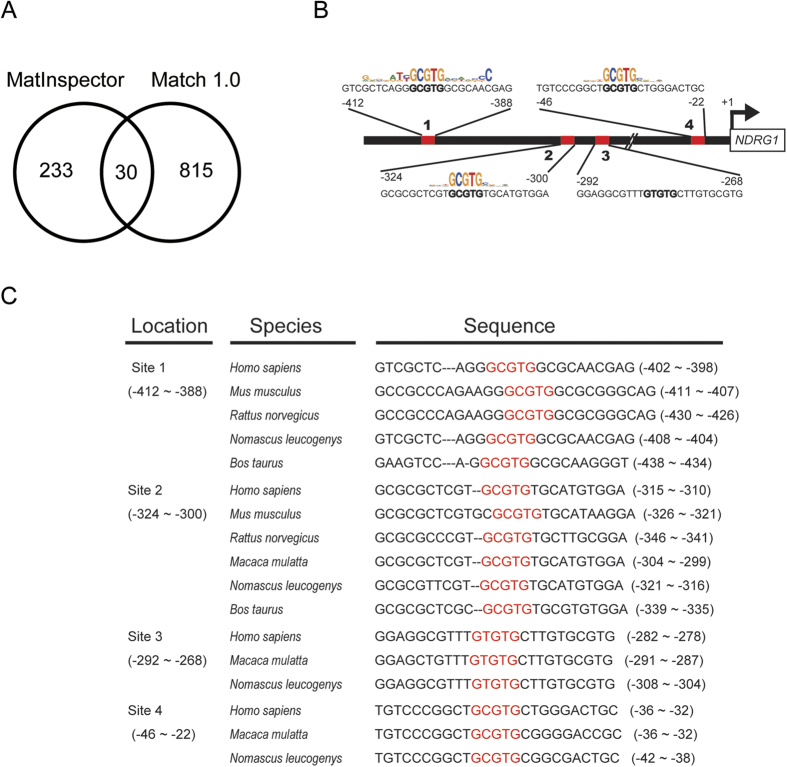
*In silico* analysis of AHR binding sites in the promoter of *NDRG1.* (**A**) Number of common transcription factor binding sites predicted by MatInspector and Match 1.0. (**B**) Locations of predicted AHR binding sites and their sequence logos in the *NDRG1* promoter. The conserved core sequence of AHR, GCGTG, is in bold. The core similarity of all sites is ≥0.75. (**C**) Conservation of the AHR binding motif among different species. The core binding motif of AHR is marked in red. The sequences of *NDRG1* (nucleotides −1,000 to + 500) in human (*Homo sapiens*), mouse (*Mus musculus*), rat (*Rattus norvegicus*), gibbon (*Nomascus leucogenys*), bovine (*Bos taurus*), and monkey (*Macaca mulatta*) were extracted from the National Center for Biotechnology Information (NCBI) database and aligned with ClustalW2.

To further investigate the regulatory mechanism of *NDRG1* in hypoxia, promoter analysis was used to identify its regulatory networks. First, *in silico* analysis using Matlnspector^37^ was conducted to search the transcription factor binding sites in the promoter region of *NDRG1*. The promoter region (−783~ + 312 bp) was determined by default using the ElDorado database^37^. Next, vertebrate matrix libraries of transcription factor binding sites with conservation profiles and core regions were used, and the core similarity was set at ≧ 0.75. We identified 81 transcription factors with 263 binding sites within the 1,095 bp promoter region. In addition, Match^TM^ 1.0 based on the Transfac 6.0 database was used to increase predictive accuracy. Using the same promoter region determined by Matlnspector, 845 transcription factor binding sites were found. As shown in Fig. 1A, there were 30 transcription factor binding sites derived from 21 transcription factors in common.

Among these common transcription factors, the AHR/ARNT heterodimer had the most predicted binding sites (n = 4). The four predicted AHR/ARNT binding sites were located at −412~−388 (site 1), −324~−300 (site 2), −292~−268 (site 3), and −46~−22 (site 4) bp (Fig. 1B). The core sequence with the highest conserved positions was 5′-GCGTG-3′. Also, we examined whether these four sites had evolutionary conservation. The *NDRG1* promoter was defined as a region of −1,000~ + 500 bp among several species, including human (*Homo sapiens*), mouse (*Mus musculus*), rat (*Rattus norvegicus*), gibbon (*Nomascus leucogenys*), bovine (*Bos taurus*), and monkey (*Macaca mulatta*). Multispecies alignment of the *NDRG1* promoter region revealed that four predicted AHR/ARNT binding sites were conserved in several species (Fig. 1C). The core sequences (5′-GCGTG-3′) of sites 1 and 2 were highly conserved in humans, rodents, and primates. Sites 3 and 4 were only conserved in primates. Based on the number of predicted binding sites and conservation among species, we chose to focus on AHR/ARNT for our experiments investigating NDGR1’s regulatory mechanisms.

**Figure 2:**
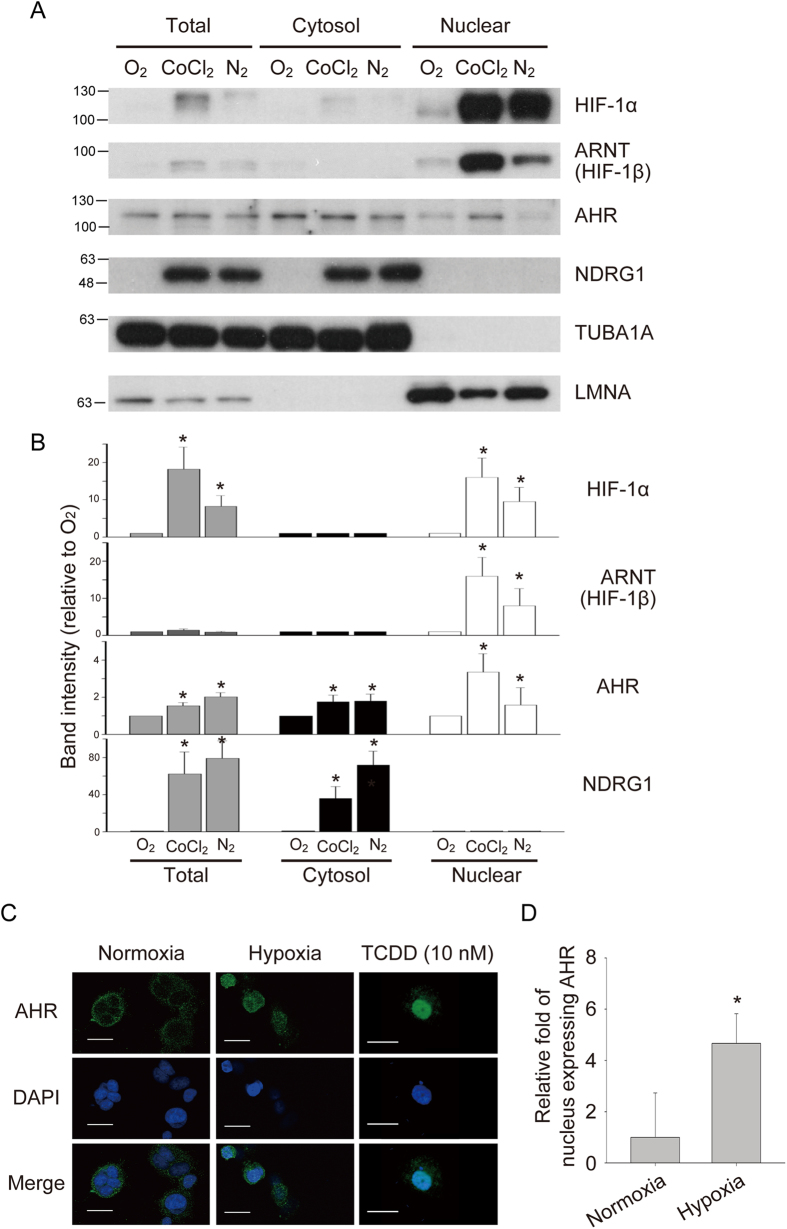
Up-regulation of nuclear AHR in hypoxia and CoCl_2_ treatment. (**A**) Western blot analysis of total lysate, cytosol fraction, and nuclear fraction of MCF-7 cells growing in normoxia (O_2_), CoCl_2_ (300 μM), and hypoxia (N_2_). α-tubulin (TUBA1A) was the loading control for cytosol fraction. Lamin A/B (LMNA) was the loading control for nuclear fraction. (**B**) Quantification of western blot analysis. Integral optical density (IOD) was calculated for each band. Data in the bar chart are the means ± SEMs from three independent experiments. *, *P* < 0.05. (**C**) Immunohistochemistry analysis of AHR in hypoxia. TCDD: 10 nM 2,3,7,8-tetrachlorodibenzodioxin, Scale bar: 25 μm. (**D**) Quantitative analysis of nuclei expressing AHR in hypoxia. Data in the bar chart are the means ± SDs from three independent experiments. *P < 0.05.

To address whether AHR/ARNT regulates *NDRG1* expression under hypoxia, the endogenous expression of AHR was first measured. Breast cancer MCF-7 cells were incubated in normoxia, in the presence of CoCl_2_ (to mimic hypoxia), or in hypoxia for 24 h. Nuclear and cytoplasmic proteins were extracted separately and examined by western blot analysis. As expected, HIF-1α and HIF-1β were significantly (*P* < 0.05) up-regulated in the nucleus under hypoxia-mimetic conditions and hypoxia (Fig. 2A,B). The amount of cytosol and nuclear AHR under hypoxia-mimetic conditions or hypoxia was also significantly (*P* < 0.05) increased (Fig. 2A,B). NDRG1 was highly expressed only in the cytoplasm of cells treated with hypoxia or CoCl_2_ (Fig. 2A,B). Immunohistochemistry analysis showed AHR could translocate to the nuclei under hypoxia and in the presence of 2,3,7,8-tetrachlorodibenzodioxin (TCDD, 10 nM) (Fig. 2C). The number of nuclei containing AHR in hypoxia was significantly (*P* < 0.05) increased (Fig. 2D). These results suggest that, under hypoxia or hypoxia-mimetic conditions, AHR/ARNT heterodimers could translocate to nucleus to induce gene transcription in the absence of xenobiotics.

**Figure 3:**
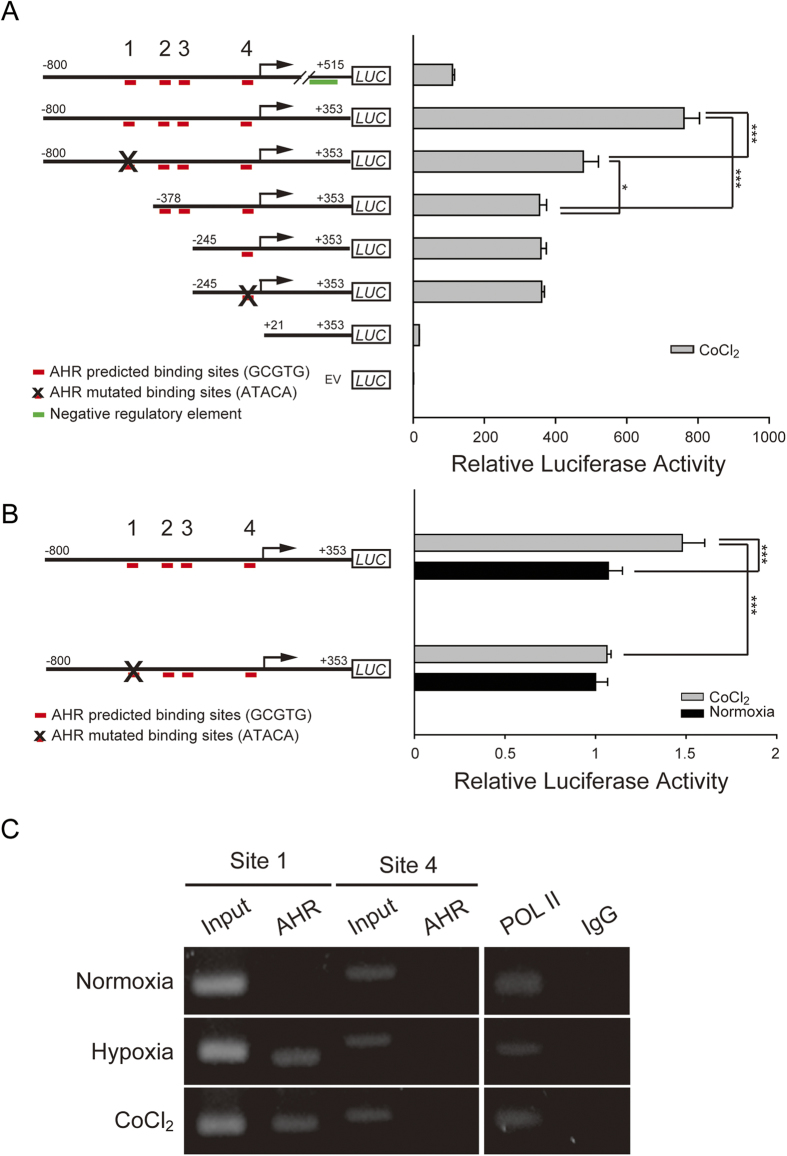
Identification of the AHR regulatory regions in the *NDRG1* promoter under CoCl_2_ treatment. (**A**) Luciferase reporter assays with serial truncation of *NDRG1* promoter. Firefly luciferase (*LUC*) was ligated to sequences with serial truncations and/or site-mutations (X mark) in the *NDRG1* promoters. Red bars indicate predicted AHR binding sites. Twenty-four hours after transfecting reporter plasmids, MCF-7 cells were treated with 300 μM CoCl_2_ for another 24 h. Firefly luciferase activities were measured and normalized to Renilla luciferase activity. EV, empty vector. *P < 0.05. ***P < 0.001. (**B**) AHR induces *NDRG1* transcription via site 1 (−412~−388 bp) under CoCl_2_ treatment. MCF-7 cells were treated by normoxia or CoCl_2_ after transfection of the reporter constructs. Data in the bar chart are the means ± SDs from three independent experiments. (**C**) Chromatin immunoprecipitation of MCF-7 cells growing under normoxia and hypoxia. Chromatin was immunoprecipitated with anti-AHR antibodies. Rabbit IgG was a negative control, and anti-polymerase II antibody was a positive control. Precipitated DNA or 1% of the chromatin input was amplified with primers for site 1 or site 4.

In order to determine whether the elevated nuclear AHR could interact with the *NDGR1* promoter through the predicted binding sites under hypoxia, a luciferase reporter assay was used to measure the promoter activity of *NDRG1*. To identify the importance of the four predicted binding sites, various truncated plasmids were constructed by fusing DNA fragments to the firefly luciferase gene (Fig. 3A, left). These DNA fragments contained a series of 5′ flanking deletions in the *NDRG1* promoter and a negative regulatory element (+353~ + 515 bp)^20^. One construct included the full −800~ + 353 bp promoter region, but had a site 1 mutation, and another shorter construct (−245~ + 353 bp) incorporated a site 4 mutation (Fig. 3A, left). A total of eight firefly luciferase reporter vectors containing various deletions or site mutations were transfected into MCF-7 cells. After transient transfection, cells were treated with CoCl_2_ for 24 h to mimic hypoxia. Transcriptional activity of the *NDRG1* promoter was quantified by measuring relative luciferase activity (Fig. 3A, right). The luciferase activities were first normalized to *Renilla* luciferase activities, and then to the value of empty vectors. As expected, when the negative regulatory element (+353~ + 515 bp) was removed, the inducible promoter activity was significantly (*P* < 0.05) increased. Subsequently, when the promoter from −800~−378 bp was removed or the AHR/ARNT predicted binding site 1 was mutated, the promoter activity was significantly (*P* < 0.05) attenuated. In contrast, the promoter activity was not affected by removal of the region containing sites 2 and 3 or by mutation at site 4. Taken together, these data suggest that site 1 (−412~−388 bp) may be an essential positive regulatory element for AHR/ARNT in *NDRG1* regulation.

In order to validate that site 1 is responsible for the hypoxia-induced promoter activity, MCF-7 cells were treated by CoCl_2_ after transfection of the luciferase reporter constructs containing the promoter region (−800~ + 353 bp), with or without the site 1 mutation. As shown in Fig. 3B, the promoter activity was strongly induced only by cobalt treatment, not in normoxia. Mutation of site 1 significantly (*P* < 0.05) abolished the promoter activity, suggesting that site 1 is specifically responsible for cobalt-induced promoter activities.

Next, we used chromatin immunoprecipitation (ChIP) to investigate the interaction between AHR and the *NDRG1* promoter (Fig. 3C). Anti-AHR antibody, anti-RNA polymerase II antibody (the positive control), and mouse IgG (the negative control) were used to precipitate protein-DNA complexes. The precipitated chromatin was amplified by PCR using primers flanking site 1 or site 4. AHR was obviously bound to site 1 of the *NDRG1* promoter in hypoxia and hypoxia-mimetic conditions, whereas no binding was observed at site 4. These data indicate that AHR positively regulates *NDRG1* transcription under hypoxic conditions through binding site 1 (−412~−388 bp).

**Figure 4:**
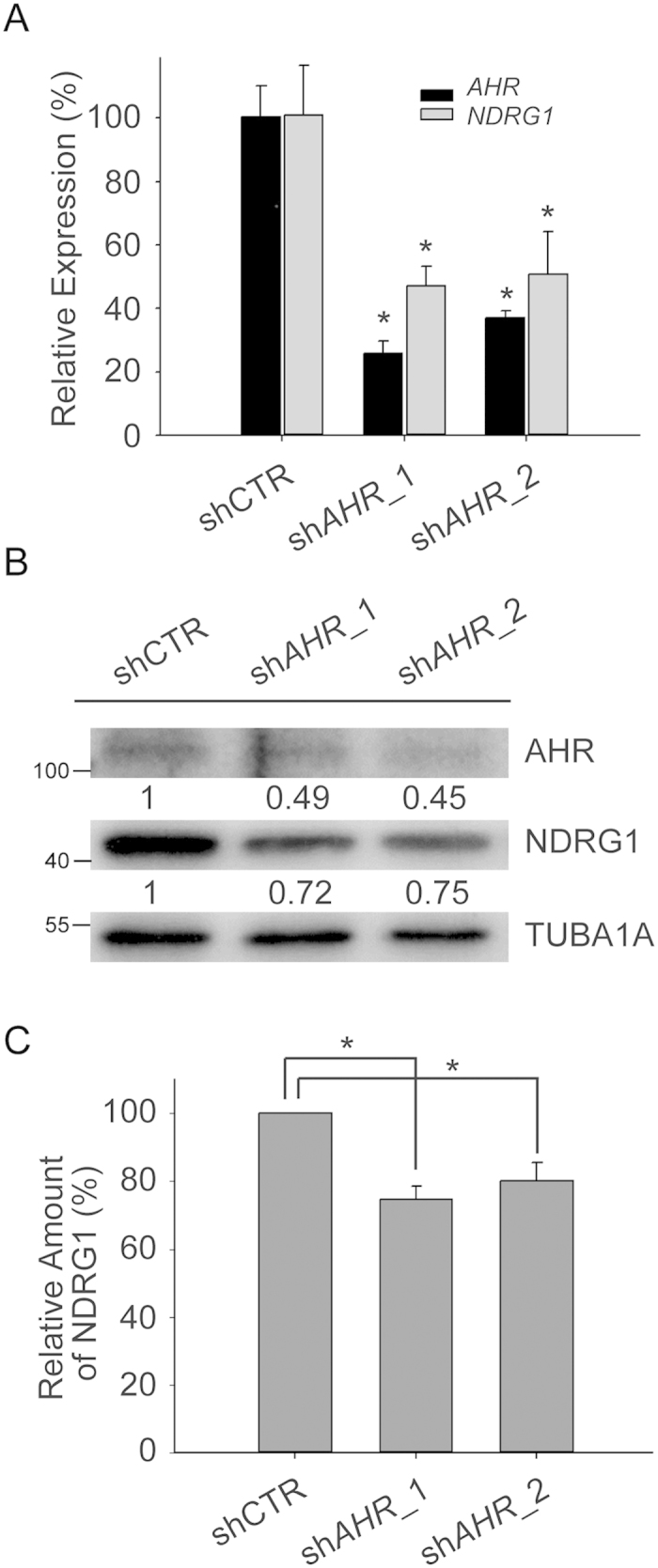
NDRG1 is activated by AHR after CoCl_2_ treatment. (**A**) Relative expression levels of *AHR* and *NDRG1* using quantitative RT-PCR. MCF-7 cells were infected with lentivirus expressing AHR-specific shRNAs (shAHR_1 or shAHR_2) or scramble control (shCTR), selected with puromycin, and then cultured in the presence of 300 μM CoCl_2_ for 24 h. *AHR* and *NDRG1* mRNA expression were detected by quantitative RT-PCR and normalized to 18S rRNA. *P < 0.05. (**B**) Western blot analysis of AHR and NDRG1 from MCF-7 cells transduced with shRNA against *AHR*. TUBA1A (α-tubulin) was the loading control. (**C**) Quantitative analysis of relative amount of NDRG1 from MCF-7 cells transduced with shRNA against *AHR*. Data in the bar chart are the means ± SDs from three independent experiments. *P < 0.05.

To confirm that AHR/ARNT heterodimers up-regulate *NDRG1* expression in hypoxia, *AHR* was knocked down in MCF-7 cells. Cells were transduced with lentivirus expressing *AHR*-specific shRNAs (sh*AHR*_1 & sh*AHR*_2) or scramble control (shCTR) and cultured in the presence of CoCl_2_ for 24 h. The knockdown efficiency of *AHR*-specific shRNAs was evaluated. Both *AHR*-specific shRNAs led to a significant (*P* < 0.05) reduction in RNA levels (Fig. 4A), and dramatically diminished AHR protein levels (Fig. 4B). Down-regulation of AHR by sh*AHR*_1 and sh*AHR*_2 both attenuated the mRNA levels of *NDRG1* (Fig. 4A) and significantly (*P* < 0.05) decreased protein levels of cobalt-induced NDRG1, indicating that *NDRG1* was regulated by AHR in hypoxia (Fig. 4B,C).

Next, we investigated the functional roles of AHR and NDRG1 in hypoxia. Since *NDRG1* was highly expressed in hypoxia, we knocked down *NDRG1* expression with lentiviral vectors expressing shRNAs. After MCF-7 cells were transduced and incubated in hypoxia, the knockdown efficiency was confirmed by quantitative RT-PCR and western blotting (Fig. S1). Both shRNAs against *NDRG1* still significantly reduced the expression of *NDRG1* mRNA (Fig. S1A) and protein (Fig. S1B) on the fifth day after transduction. However, since the knockdown efficiency of sh*NDRG1*_2 was more effective, we chose to use sh*NDRG1_*2 for subsequent experiments.

**Figure 5:**
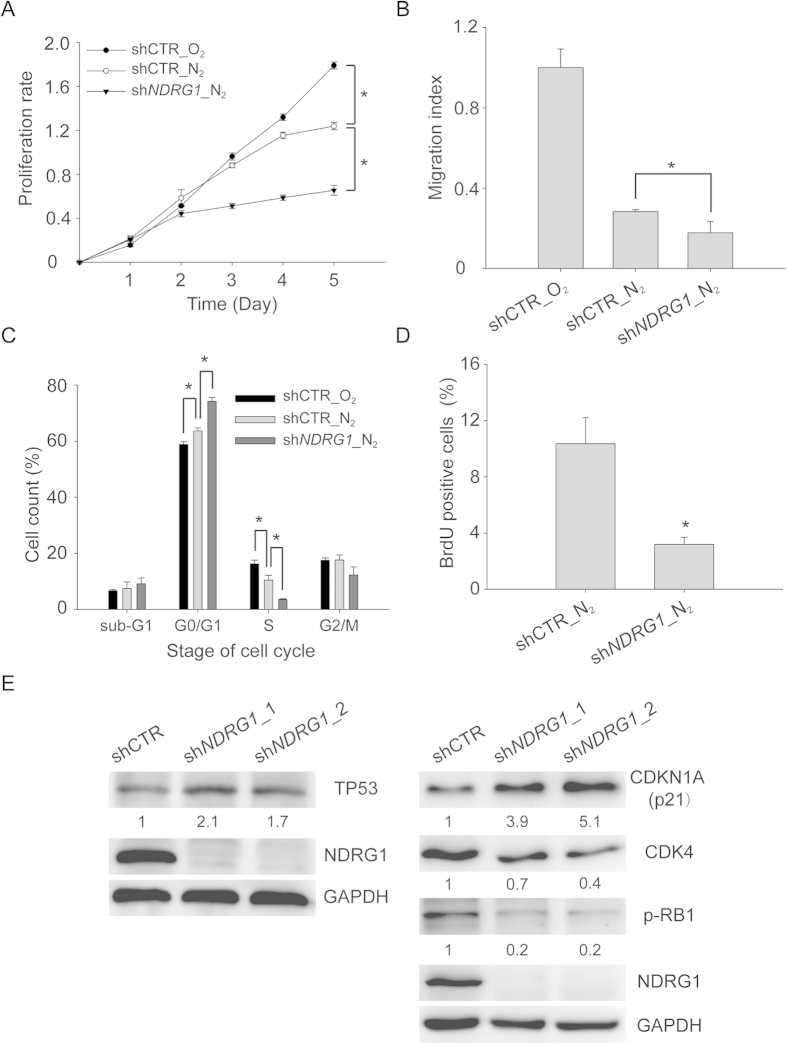
Knockdown of *NDRG1* suppresses cell growth, inhibits cell migration, and induces cell cycle arrest under hypoxia. (**A**) Cell proliferation using CCK-8 assay. The rates of proliferation were normalized to the absorbance on day 0. (**B**) Transwell migration assay. Cells were measured for migration ability after 36 h of inoculation. (**C**) Quantitative analysis of cell cycle distribution by flow cytometry. Cells were harvested and stained with propidium iodide after incubation under normoxic or hypoxic conditions for 3 days. (**D**) Quantitative analysis of BrdU-positive cells by flow cytometry. Cells were incubated with BrdU for 30 min and stained with FITC-labeled anti-BrdU antibody and 7-AAD after 3d of hypoxia. (**E**) Western blot analysis of G1-related proteins in *NDRG1* knockdown and control cells. Protein from whole cell lysates was blotted with TP53, CDKN1A (p21), CDK4, and p-RB1 (Rb) and NDRG1 antibodies after hypoxic incubation for 3 days. GAPDH was the loading control. All experiments in panels A-D were repeated at least three times, and the results are the means ± SDs. *P < 0.05.

CCK-8 assays were performed to examine the effect of *NDRG1* on cell proliferation in hypoxia. As expected, cells grew slower in hypoxia as compared to normoxia (Fig. 5A). Knockdown of *NDRG1* further suppressed cell growth under hypoxia (Fig. 5A). We also investigated the cell migration ability using transwell migration assays. The migration assays were finished in two days. Within this time period, there is no difference in proliferation (Fig. 5A), but the motility of MCF-7 cells was decreased when *NDRG1* was knocked down under hypoxia (Fig. 5B). Similarly, normoxic cells exhibited more migration ability than hypoxic cells.

Based on the significantly decreased proliferation rate in *NDRG1* knockdown cells from day 3 (Fig. 5A), we checked its effect on the cell cycle. Unsurprisingly, *NDRG1* knockdown cells accumulated in G0/G1 phase, with few cells in S phase (Fig. 5C). This phenomenon was validated by examining the effect of BrdU incorporation. Knockdown of *NDRG1* decreased the percentage of BrdU positive cells (S phase cells) under hypoxia (Fig. 5D). Also, since hypoxia could induce cell cycle arrest at G1 phase through up-regulation of cyclin-dependent kinase inhibitors (CDKIs) and reduction of CDK and p-RB protein levels^38^, western blot analyses showed that knockdown of *NDRG1* led to elevation of TP53 and CDKN1A (p21) proteins, and reduction of CDK4 and p-RB proteins (Fig. 5E). Taken together, these data suggest that *NDRG1* knockdown in MCF-7 cells induces p53-mediated G1 arrest under hypoxia.

**Figure 6:**
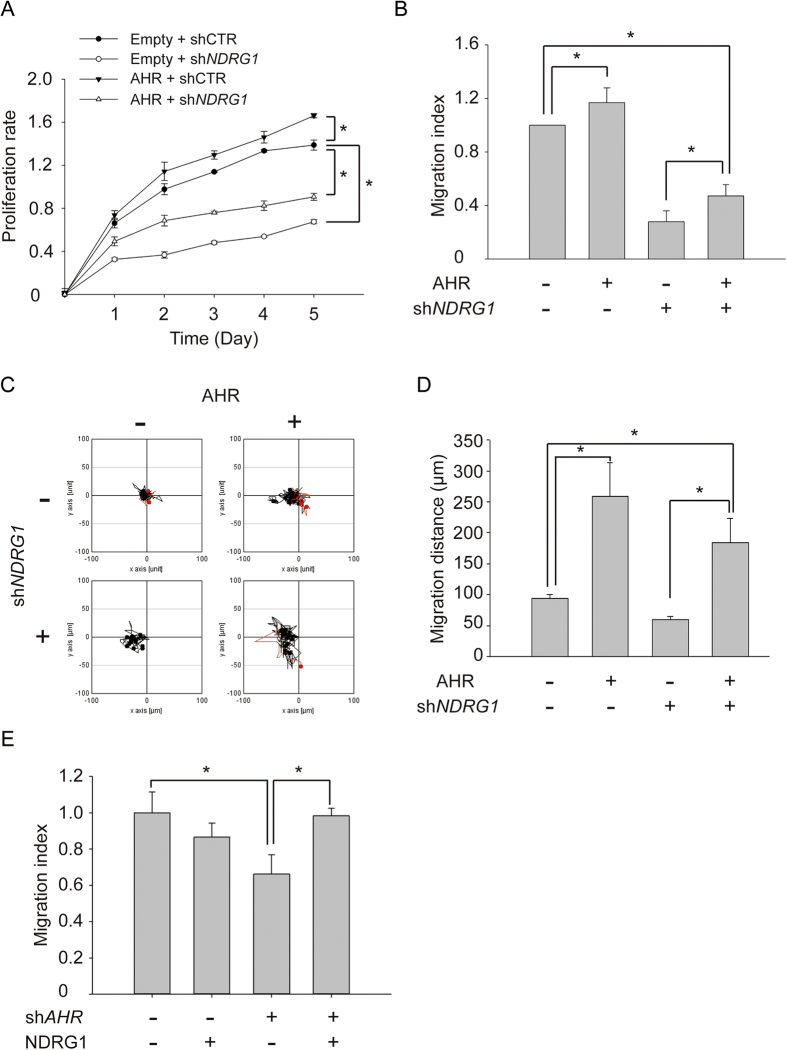
AHR increases cell growth and cell migration under hypoxia through targeting NDRG1. (**A**) Cell proliferation using the CCK-8 assay. Growth curves were analyzed in MCF-7 cells knocked down for *NDRG1* and/or overexpressing *AHR*. The proliferation rate was normalized to the absorbance on day 0. (**B**) Transwell migration assay. Migration ability of each group was measured at 24 h after seeding. (**C**) Migration ability in hypoxic mimic condition using time-lapse video microscopy. Representative graph shows motile activity of 15 randomly chosen cells. Migration traces to the right are shown in red, and to the left in black. (**D**) Quantitation of migration distance over 16 h. (**E**) Transwell migration assay. Migration ability of each group was measured at 24 h after seeding. Cell motility was analyzed in MCF-7 cells knocked down for *AHR* and/or overexpressing *NDRG1*. All experiments were repeated at least three times, and the results are the means ± SDs. *P < 0.05.

Because of the evidence that AHR positively regulates the expression of *NDRG1* during hypoxia (Figs 3 and 4), we hypothesized that overexpression of AHR could enhance the functions of NDRG1 under hypoxia. Thus, we established MCF-7 cells stably overexpressing AHR. Cells were transfected with pcDNA3-*AHR*, and selected with G418 for two weeks. The expression levels of AHR in AHR overexpressing cells were confirmed by western blot analysis (Fig. S2). Next, the stably overexpressing or control cells were further knocked down for *NDRG1* expression by RNA interference (shRNA) and then cultured in hypoxia. The effects on cell proliferation were examined by CCK-8 assays. The results showed that overexpression of AHR significantly (*P* < 0.05) facilitated cell growth under hypoxia as compared with empty vector controls (Fig. 6A). Similarly, knockdown of *NDRG1* significantly (*P* < 0.05) attenuated cell proliferation under hypoxia, and the phenomenon can be rescued by overexpressing AHR (Fig. 6A). Moreover, using transwell migration assay and time-lapse video microscopy, the results showed that overexpression of AHR significantly (*P* < 0.05) increased and *NDRG1* knockdown significantly (*P* < 0.05) decreased the motility of MCF-7 cells under hypoxia (Fig. 6B–D). Conversely, *AHR* knockdown significantly decreased the motility of MCF-7 cells under hypoxia, and this phenomenon was rescued by overexpressing *NDRG1* (Fig. 6E). These data reveal that AHR may facilitate cell growth and cell migration under hypoxia by up-regulating *NDRG1*.

## Discussion

In this study, we demonstrated that AHR directly up-regulates *NDRG1* under hypoxia in MCF-7 breast cancer cells. First, we showed that the enhanced nuclear protein levels of AHR were observed under hypoxia. Next, luciferase reporter assays indicated that the AHR binding site at −412~−388 bp was correlated with transcriptional activation of *NDRG1*. Finally, functional assays showed that AHR could facilitate cell proliferation and motility under hypoxia via regulating *NDRG1*.

Previously, our studies indicated that *NDRG1* plays a critical role in tumor adaptation to oxygen fluctuations^39^. Here we provided the first evidence that AHR mediates cobalt-mimetic hypoxia induction of *NDRG1* through an AHR/ARNT binding site in the promoter using various promoter truncations or site-mutation constructs.

It has been reported that *NDRG1* is regulated by several hypoxia-responsive transcription factors, such as HIF-1α^18^, HIF-2α^40^, and EGR-1/SP1^20^. Among these transcription factors, HIF-1α is the most crucial for *NDRG1* transcription. Owing to the fact that ARNT is crucial for both AHR and HIF-1α, several studies have suggested that cross-talk exists and affects the regulation of gene expression. Some studies showed a competitive effect between the two pathways due to sharing the same heterodimerization partner (ARNT)^31^,^33^,^41^. However, we observed an additive cross-talk between the AHR and HIF-1 signaling pathways on account of containing separate HREs and XREs in the *NDRG1* promoter. Similarly, a previous report showed that erythropoietin, containing both HREs and XREs in its promoter, had enhanced expression levels under hypoxia via activation of AHR^31^.

It is well known that AHR serves as a ligand-activated transcription factor in response to the toxic effect of xenobiotics, including polycyclic and halogenated aromatic hydrocarbons^22^. However, our results showed that AHR translocated to nuclei and bound to the *NDRG1* promoter during hypoxia (Figs 2C and 3C). Hence we supposed that AHR could active *NDRG1* expression under hypoxia through a ligand-independent mechanism and that the Hsp90-associated co-chaperone p23 might participate in this mechanism. A previous study has shown that AHR could be activated by dissociating with p23 in a ligand-independent manner^42^. Furthermore, hypoxia was shown to trigger p23 cleavage, resulting in abolishment of the function of p23^43^. Collectively, these results imply that hypoxic conditions might induce ligand-independent activation of AHR through p23 cleavage. However, this mechanism should be validated in future work.

AHR plays an important role in the stages of tumor development, such as proliferation, apoptosis, and metastasis^36^,^44^. We observed that ectopic expression of *AHR* enhanced cell proliferation and migration in hypoxic MCF-7 cells; conversely, these functional effects could be significantly reduced while knocking down *NDRG1* (Fig. 6). We attributed this functional effect to the fact that AHR increased the expression of *NDRG1* under hypoxia. Previous studies indicated that knockdown of *AHR* could attenuate cell growth and induce cell cycle arrest at G1 phase in MCF-7 and MDA-MB-231 cells^36^. Similarly, our results showed that knockdown of *NDRG1* led to accumulation of cells in G1 phase and attenuation of cell growth and motility under hypoxia (Fig. 5C,E). These results were consistent with previous reports indicating that *NDRG1* facilitated cell survival and migration under hypoxic stress^45^. Furthermore, it was known that hypoxia could induce cell cycle arrest at G1 phase through up-regulation of cyclin-dependent kinase inhibitors (CDKIs) and reduction of CDK and p-RB protein levels^38^. Previous studies also indicated that NDRG1 could suppress GSK-3β activity, which plays a key regulatory role in controlling G1-related proteins in hypoxia^17^,^38^. Indeed, our results demonstrated that knockdown of *NDRG1* led to elevation of TP53 and CDKN1A (p21) proteins and reduction of CDK4 and p-RB proteins (Fig. 5E). All these data suggest that AHR and NDRG1 are involved in the same signaling pathway.

## Methods

### Cell culture and reagents

Breast adenocarcinoma MCF-7 cells were maintained in Dulbecco’s modified Eagle’s medium (DMEM; GIBCO, Carlsbad, CA, USA) containing 10% fetal bovine serum (FBS) and 1% antibiotics (penicillin-streptomycin–amphotericin solution; Biological Industries, Beit-Haemek, Israel). All cells were maintained at 37 °C in a humidified incubator with 5% CO_2_ and 95% air. Hypoxic cells were incubated in a hypoxia chamber (InVivO2-200; Ruskinn Technology, Leeds, UK) with 0.5% O_2_, 5% CO_2_ and 94.5% N_2_ for 24 h. Alternatively, cells were treated with 300 μM CoCl_2_ (CoCl_2_; Sigma, St. Louis, MO, USA) for 24 h to mimic hypoxia.

### Prediction of transcription factor binding sites

MatInspector, a Genomatix program (http://www.genomatix.de/) using the weighted matrix-based database Matbase, was used for predicting transcription factor binding sites. The promoter sequence was selected from Genomatix’s ElDorado database. In addition, another weighted matrix-based program, Match public version 1.0 using weight matrices from TRANSFAC^®^ Public 6.0, (http://www.gene-regulation.com/cgi-bin/pub/programs/match/bin/match.cgi), was used.

### *NDRG1* promoter constructs

The *NDRG1* promoter region (−800~ + 515 bp) was amplified from human genomic DNA. The primers with *Mlu*I and *Hind*III restriction sites are listed in Table 1. The PCR product was cloned into the pJET1.2/blunt cloning vector using the CloneJET PCR cloning kit (Fermentas, Thermo Scientific, Waltham, MA, USA). The *NDRG1* promoter fragment was then digested with *Mlu*I and *Hind*III (TaKaRa, Shiga, Japan), and subcloned into the pGL3 Basic luciferase vector (Promega, Madison, WI, USA). Serial 5′-deleted *NDRG1* promoters prepared by PCR amplification using forward primers containing *Mlu*I restriction sites and reverse primers containing *Hind*III restriction site are listed in Table 1. The truncated *NDRG1* promoter fragments were all subcloned into the pGL3 Basic luciferase vector at the *Mlu*I/*Hind*III sites.

### Site-directed mutagenesis

To mutate the core binding site (5′-GCGTG-3′), two primer pairs were provided as shown in Table 1. KAPA HiFi HotStart (KAPA Biosystems, Wilmington, MA, USA) was used to replicate both plasmid strands with designed primer pairs. The newly constructed plasmids were prepared by digestion of the parental strands by *Dpn*I endonuclease (New England Biolabs, Inc., Ipswich, MA, USA), transformation, and plasmid extraction. Mutated sequences were validated by sequencing.

### Luciferase reporter assay

MCF-7 cells were seeded in 24-well plates at a density of 1 × 10^5^ cells/well and transfected the next day using Lipofectamine LTX (Invitrogen, Carlsbad, CA, USA) in Opti-MEM (GIBCO). For transient transfection, 1 μg of reporter constructs was added. To control transfection efficiency, cells were cotransfected with 0.1 μg pGL4.74 [hRluc/TK] plasmid. Six hours after transfection, cells were incubated in fresh complete medium and kept at 37 °C for 18 h. Cells were then treated with 300 μM CoCl_2_ for 24 h to mimic hypoxia. After being lysed in cell lysis buffer, the luciferase activity was performed using the Dual-Glo luciferase reporter assay system (Promega).

### Quantitative RT-PCR

Total RNA was extracted by TRIzol reagent (Life Technologies) according to the manufacturer’s protocol. One μg of total RNA was reverse-transcribed using the High-Capacity cDNA Reverse Transcription Kit (Applied Biosystems). Ten percent of each reaction was used as a template for quantitative real-time PCR with FastStart Universal SYBR Green Master (Roche Diagnostics, Branchburg, NJ, USA) and performed on an ABI 7900HT instrument. Reactions were done in triplicate and expression was normalized to 18S rRNA. The primer pairs used for detection of cDNAs are listed in Table 1.

### RNA interference via lentivirus

The lentiviral vector pLKO.1 expressing silencing shRNAs against *AHR* and *NDRG1*, and nonsilencing control pLKO.1 null-T, were purchased from the National RNAi Core Facility of Academia Sinica (Taipei, Taiwan). Lentiviruses were made by cotransfection into HEK293T cells with the lentiviral packaging plasmid (psPAX2), envelope plasmid (pMD2.G) and shRNA-containing plasmid. Virus-containing supernatants were collected at 24, 48 and 72 h after transfection and filtered through a 0.22 μm syringe filter (Millipore, Billerica, MA, USA). MCF-7 cells were infected twice in the presence of 8 μg/mL polybrene (Invitrogen) and selected with 2 μg/ml of puromycin (Sigma) for 3 days.

### Western blot analysis

Total cell lysates or nuclear extracts were prepared and proteins were separated using SDS-PAGE (10%). Proteins in the gel were transferred electrophoretically to polyvinylidene difluoride membranes (Bio-Rad Laboratories, Hercules, CA, USA). The membranes were blocked in 5% milk and immunoblotted with antibodies against the following proteins, including HIF-1α (Millipore), ARNT (HIF-1β) (Abcam Inc., Cambridge, MA, USA), AHR (GeneTex, Irvine, CA, USA), TP53 (Dako, Copenhagen, Denmark), CDKN1A (p21) (GeneTex), CDK4 (GeneTex), p-RB1 (GeneTex), GAPDH (GeneTex), NDRG1 (Abcam), TUBA1A (α-tubulin) (Novus Biologicals, Littleton, CO, USA) and LMNA (Lamin A/B) (Novus). Bound primary antibodies were reacted with horseradish peroxidase-conjugated goat anti-rabbit IgG or rabbit anti-mouse IgG (GeneTex) and detected with the chemiluminescent western blotting system (Millipore).

### Immunofluorescence

MCF-7 cells were plated on glass coverslips in 24-well plates at 60% confluence. After seeding overnight, cells were cultured under normoxic or hypoxic conditions for 24 h. The cells were fixed in 4% paraformaldehyde (Sigma) at room temperature for 10 min, permeabilized, and blocked with phosphate buffered saline (PBS) containing 10% normal goat serum (GIBCO) and 0.2% Tritin X-100 (Sigma) for 1 h. The cells were then washed and incubated with AHR antibody (GeneTex) at 4 °C overnight followed by incubation with Alexa Fluor 488-conjugated secondary antibody (Molecular Probes, Eugene, OR, USA). Nuclei were stained with 4′, 6-diamidino-2-phenylindole (DAPI) (Sigma). Coverslips were mounted on glass slides using Aqua-Poly/Mount (Thermo Fisher Scientific, Hudson, MA, USA). MCF-7 cells treated with 2,3,7,8-tetrachlorodibenzodioxin (TCDD, 10 nM) was served as a positive control of AHR nuclear translocation. The fluorescence images were viewed using a Leica SP5 confocal microscope (Leica Microsystems, Wetzlar, Germany).

### Chromatin immunoprecipitation (ChIP)

ChIP assays were performed according to the manufacturer’s instructions (Pierce/Thermo Scientific, Rockford, IL, USA). Cells were cross-linked in 1% formaldehyde for 10 min at room temperature, washed with cold PBS, and harvested into tubes. After lysing cells, the genomic DNA was digested with micrococcal nuclease to generate DNA fragments of 200–500 bp. The fragmented chromatin was used for immunoprecipitation by incubating overnight at 4 °C with AHR antibodies (GeneTex), RNA polymerase II antibodies (positive control), or normal mouse IgG (negative control). ChIP Grade Protein A/G Plus Agarose (Santa Cruz Biotechnology, Shanghai, China) was added for a further 1 h incubation at 4 °C. After washing, the cross-links were digested by treating with proteinase K for 1.5 h at 65 °C. DNA samples were purified using a DNA Clean-Up Column (Qiagen) and analyzed by PCR using the primer pairs listed in Table 1.

### Transwell migration assay

Cells (1.5 × 10^5^ cells in 200 μl of serum-free medium) were added to the upper chamber of a Transwell unit (Corning Inc., Corning, NY, USA). The lower chamber was filled with 600 μl DMEM containing 10% FBS. After incubation, cells were fixed in 600 μl with 75% methanol/25% acetic acid (Sigma) for 20 min and stained with 0.1% crystal violet for another 20 min. Cells on the upper side of the membrane surface were removed by scraping with a cotton swab, and the cells that passed through the filter were destained using 10% acetic acid (Sigma). The absorbance was measured at 570 nm with an ELISA reader (BioTek, Winooski, VT, USA).

### Cell cycle analysis

Cells were harvested by trypsinization, washed with PBS, and fixed with cold 100% ethanol at −20 °C overnight. Thereafter, cells were washed twice and resuspended in PBS containing 20 μg/ml propidium iodide (Life Technologies), 0.1% triton-X-100 (Sigma) and 100 μg/ml RNase A (Sigma) for 30 min. The suspension was passed through a nylon mesh filter and analyzed using a Beckman Coulter FC500 (Beckman, Brea, CA, USA) and CXP Analysis software.

### BrdU incorporation assay

Analysis of BrdU incorporation was performed using the FITC BrdU Flow Kit (BD Biosciences, San Jose, CA, USA) according to the manufacturer’s protocol. MCF-7 cells were incubated in culture medium with 10 μM BrdU for 30 min. Then the cells were trypsinized, washed, fixed and permeabilized with Cytofix/Cytoperm, and treated with DNase to expose incorporated BrdU. The incorporated BrdU was stained with FITC-conjugated anti-BrdU and total DNA was labeled with 7-amino-actinomycin D. After staining, cells were resuspended in staining buffer and analyzed on a Beckman Coulter FC500 (Beckman).

## Additional Information

**How to cite this article**: Li, E.-Y. *et al.* Aryl Hydrocarbon Receptor Activates *NDRG1* Transcription under Hypoxia in Breast Cancer Cells. *Sci. Rep.* **6**, 20808; doi: 10.1038/srep20808 (2016).

## Supplementary Material

Supplementary Information

## Figures and Tables

**Table 1:** Primer list.

**mRNA/gene promoter**	**Sequence (5**′**–3**′)	**Experimental use**
*NDRG1* promoter (−800~ + 515 bp)	5′-ACGCGTTGCAGGCCAATAGTTACGCA-3′	Promoter constructs
	5′-AAGCTTTGGGATTCGGAGACGACGCCA-3′	
*NDRG1* promoter (−378~ + 353 bp)	5′-ACGCGTGAAACTCTGAGGCAGAGAT-3′	Promoter constructs
	5′-AAGCTTGGTGTCTGCACCCAAAGC-3′	
*NDRG1* promoter (−245~ + 353 bp)	5′-ACGCGTCTGCACAGGCCGAG-3′	Promoter constructs
	5′-AAGCTTGGTGTCTGCACCCAAAGC-3′	
*NDRG1* promoter ( + 21~ + 353 bp)	5′-ACGCGTCCTATAAAGTCGCCCTCC-3′	Promoter constructs
	5′-AAGCTTGGTGTCTGCACCCAAAGC-3′	
*NDRG1* promoter (−57~−13 bp)	5′-GGAAGGGGTGTGTCCCGGCTATACACTGGGACTGCGAGGGTCTGG-3′†	Site-directed mutagenesis
	5′-CCAGACCCTCGCAGTCCCAGTGTATTGCCGGGACACACCCCTTCC-3′†	
*NDRG1* promoter (−424~−378 bp)	5′-GGAAGTGAAGGGAGTCGCTCAGGATACAGCGCAACGAGACTCTTAGAAG-3′†	Site-directed mutagenesis
	5′-CTTCTAAGAGTCTCGTTGCGCTGTATCCTGAGCGACTCCCTTCACTTCC-3′†	
*AHR*	5′-CCCATATCCGAATGATTAAGACTG-3′	Real-time RT-PCR
	5′-CGTAAATGCTCTGTTCCTTCC-3′	
*NDRG1*	5′-GGCAACCTGCACCTGTTCATCAAT-3′	Real-time RT-PCR
	5′-TGAGGAGAGTGGTCTTTGTTGGGT-3′	
18S rRNA	5′-TCAACTTTCGATGGTAGTCGCCGT-3′	Real-time RT-PCR
	5′-TCCTTGGATGTGGTAGCCGTTTCT-3′	
*NDRG1* site 1	5′-CATGGCGACACGGAAGGCTGAGAATCGG-3′	ChIP
	5′-CATTTGTGTCTCCGTATGGGCGGAGGCC-3′	
*NDRG1* site 4	5′-CATCCTACGACTGCTTGCGCAACAG-3′	ChIP
	5′-CATAGGCGAGGTTTGTTTACGTCCGG-3′	

^†^AHR mutated binding sties were underlined.
