# Supplementary material for: Aryl Hydrocarbon Receptor Activates NDRG1 Transcription under Hypoxia in Breast Cancer Cells
Source: Sci Rep. 2016 Feb 8;6:20808. doi: 10.1038/srep20808 (PMC4745107; doi:10.1038/srep20808)
Supplement: Supplementary Information [file srep20808-s1.doc]

Aryl Hydrocarbon Receptor Activates *NDRG1* Transcription under Hypoxia in Breast Cancer Cells

En-Yu Li^†^, Wei-Yung Huang^†^, Ya-Chu Chang, Mong-Hsun Tsai, Eric Y. Chuang, Qian-Yu Kuok, Shih-Ting Bai, Lo-Yun Chao, Yuh-Pyng Sher^*^, Liang-Chuan Lai^*^


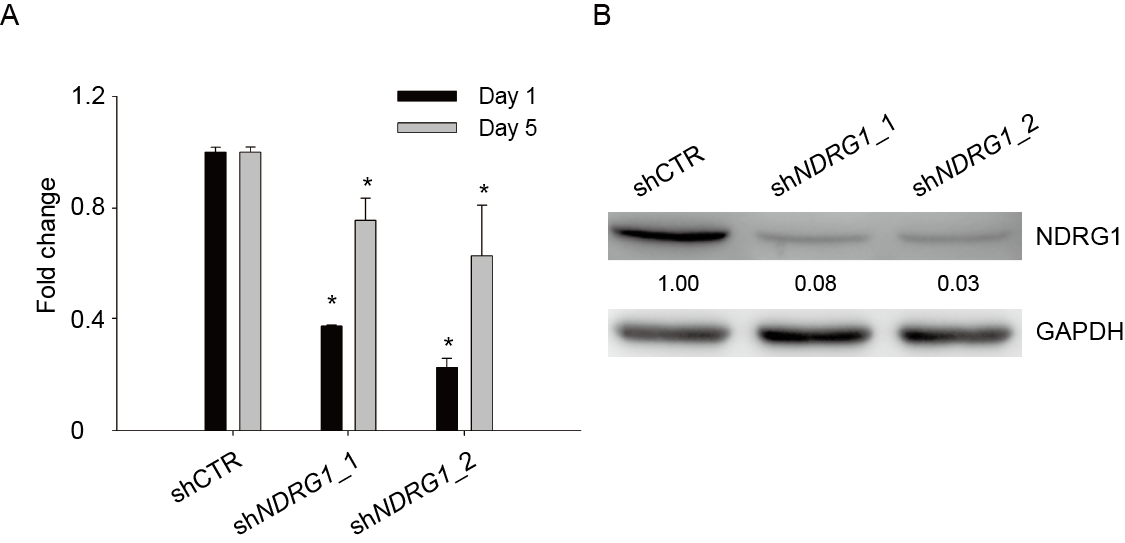


Figure S1. Knockdown of *NDRG1* in the MCF-7 cells using shRNA. (A) Relative transcriptional levels of *NDRG1* after 1 and 5 days of transduction. Cells were first transduced by lentivirus expressing *NDRG1*-specific shRNAs (shNDRG1_1 or shNDRG1_2) or scramble control (shCTR), selected with puromycin, and then cultured under hypoxia. NDRG1 mRNA expression was detected by quantitative RT-PCR and normalized to 18S rRNA. Data in the bar chart are the means ± SDs from three independent experiments. *, *P*<0.05. (B) Immunoblotting of NDRG1 after 5 days of transduction. GAPDH was the loading control.


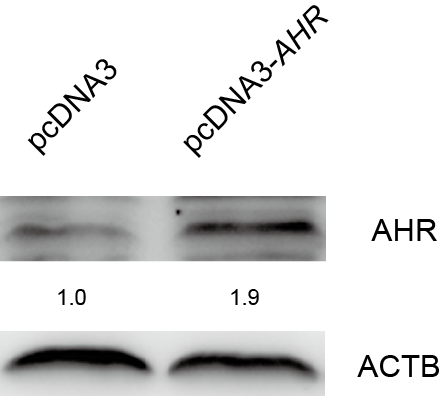


Figure S2. Overexpression of AHR in MCF-7 cells. Cells were transfected with the AHR expression construct (pcDNA3-AHR) or empty vector (pcDNA3) and selected with G418 for 2 weeks. ACTB (β-actin) was served as a loading control.
